# Labyrinthine Fistula in Cholesteatoma Patients: Outcomes of Partial Labyrinthectomy With “Underwater Technique” to Preserve Hearing

**DOI:** 10.3389/fneur.2022.804915

**Published:** 2022-03-02

**Authors:** Annalisa Pace, Alessandro Milani, Daniela Messineo, Valeria Rossetti, Salvatore Cocuzza, Antonino Maniaci, Claudio Vicini, Giannicola Iannella, Giuseppe Magliulo

**Affiliations:** ^1^Organi di Senso Department, Sapienza University of Rome, Rome, Italy; ^2^Scienze Chirurgiche Department, Sapienza University of Rome, Rome, Italy; ^3^Radiological, Oncological and Anatomo-Pathological Sciences Department, Sapienza University of Rome, Rome, Italy; ^4^Otorinolaringoiatria Department, University of Catania, Catania, Italy; ^5^Otolaryngology, Head-Neck and Oral Surgery Unit, Department of Head-Neck Surgery, Morgagni Pierantoni Hospital, Forlì, Italy

**Keywords:** labyrinth diseases, fistula, cholesteatoma, otologic surgical procedure, underwater technique

## Abstract

Labyrinthine fistula (LF) is one of the most important complications of cholesteatoma and is defined as an abnormal communication between the inner and the middle ear. This study aims to describe our experience with the partial labyrinthectomy evaluating the post-operative hearing results. Twenty-one patients who presented labyrinthine fistula in the semicircular canals were included in the present study. Hearing impairment was present in 48% of patients (10/21). A pre-operative assessment using the Gardner–Robertson hearing classification showed the following: 52%, Class I; and 48%, Class II. A post-surgical Gardner–Robertson hearing classification evidenced the following: 43%, Class I; and 57%, Class II. The presence of LF is usually considered a negative prognostic factor for hearing preservation. The key point of partial labyrinthectomy surgery is the preservation of structures, keeping them wet with Ringer's solution throughout the procedures, and not performing suction that is close to the opened LF. The bony labyrinth is drilled underwater without suction, removing the entire cholesteatoma matrix and quickly plugging the site before and after the LF. This faster plugging of the labyrinth makes it possible to preserve the peri-lymph and the endo-lymph fluid and the hearing function. This study showed that a partial labyrinthectomy is useful for maintaining serviceable hearing in patients with LF.

## Introduction

Labyrinthine fistula (LF) is a pathological condition in which an abnormal communication between the inner ear and the middle/mastoid cavity is present (1). It is mainly a complication of cholesteatoma due to erosion of the bony covering of the posterior labyrinth leading to the exposure of the membranous one (2).

In patients with cholesteatoma, CT and MR are the best methods for diagnosing and planning surgical management.

Surgical management is, in fact, the treatment of choice in terms of hearing preservation, although which technique should be performed is a source of debate.

Some authors retain the cholesteatoma matrix on the side of the LF to avoid damaging the cochlear function. Others claim to manage them by totally removing the cholesteatoma and repairing the labyrinth using bone sealing (3, 4). In cases of cholesteatoma matrix removal, the fistula is commonly covered without suctioning and without drilling the bone labyrinth to reduce the risk of hearing damage.

In 2008, Magliulo et al. proposed a partial labyrinthectomy as a safe technique for treating LF and for preserving the hearing, using a total removal of the cholesteatoma matrix and the involved bony covering (5). This procedure may also be used in petrous bone cholesteatoma for its ability to preserve the anatomical structure and the functional activity of the labyrinth (6).

This study aims to describe our experience with the partial labyrinthectomy over the last 12 years, evaluating the obtained post-operative hearing results and demonstrating the preservation of the labyrinthine fluids *via* MR sequences.

## Materials and Methods

The data of 264 patients, who are surgically treated for cholesteatoma, were extracted from a database covering the period from 2007 to 2020. Twenty-one patients, who presented labyrinthine fistula in the semicircular canals, were included in the present study. Patients with fistula of the cochlea or vestibule were excluded. All patients were treated by a senior surgeon (GM) *via* partial labyrinthectomy and hearing preservation technique. This research was performed following the principle of the Declaration of Helsinki and approved by the local ethics committee of the University “Sapienza,” Rome.

### Patient Characteristics

The database was analyzed for presenting the signs and symptoms, surgical details, and post-operative complications.

The hearing status was assessed with pure tone audiometry (PTA) and speech discrimination. The degrees of hearing preservation and loss were defined according to the Gardner–Robertson classification system. Only the patients in Classes I–II, with possible hearing preservation, were enrolled.

A pre-operative CT showed the extension of the disease and confirmed the presence of LF.

Surgical records were cataloged according to the Dornhoffer and Milewski classification, and only the patients in Class II were included (7).

### Surgical Management

The chosen surgical technique was the subtotal petrosectomy, in view of the extension of the cholesteatoma. All definitive surgical procedures were performed by the senior author (GM).

In all cases, a partial labyrinthectomy, as described by Magliulo et al. in 2008, was performed with an exclusive microscopic approach (5).

During a partial labyrinthectomy, the cholesteatoma matrix was radically removed, isolating it from the entire circumference of the LF. Firstly, the membranous labyrinth with the matrix was excised using a sickle knife, and then, the opening in the labyrinth was rapidly closed using a bone wax. While it remained open, the membranous labyrinth was kept wet with a Ringer's solution, and no suction was applied close to the openings of the semicircular canals to protect the anatomy and the function of the membranous labyrinth of the vestibule. Ringer's solution was firstly administered with an irrigation tube, thus, developing a pool. After that, it was kept wet through irrigation when drilling the bone. There is no specific amount of water, but the area should always be wet and viewable through the water.

**Figure 1:**
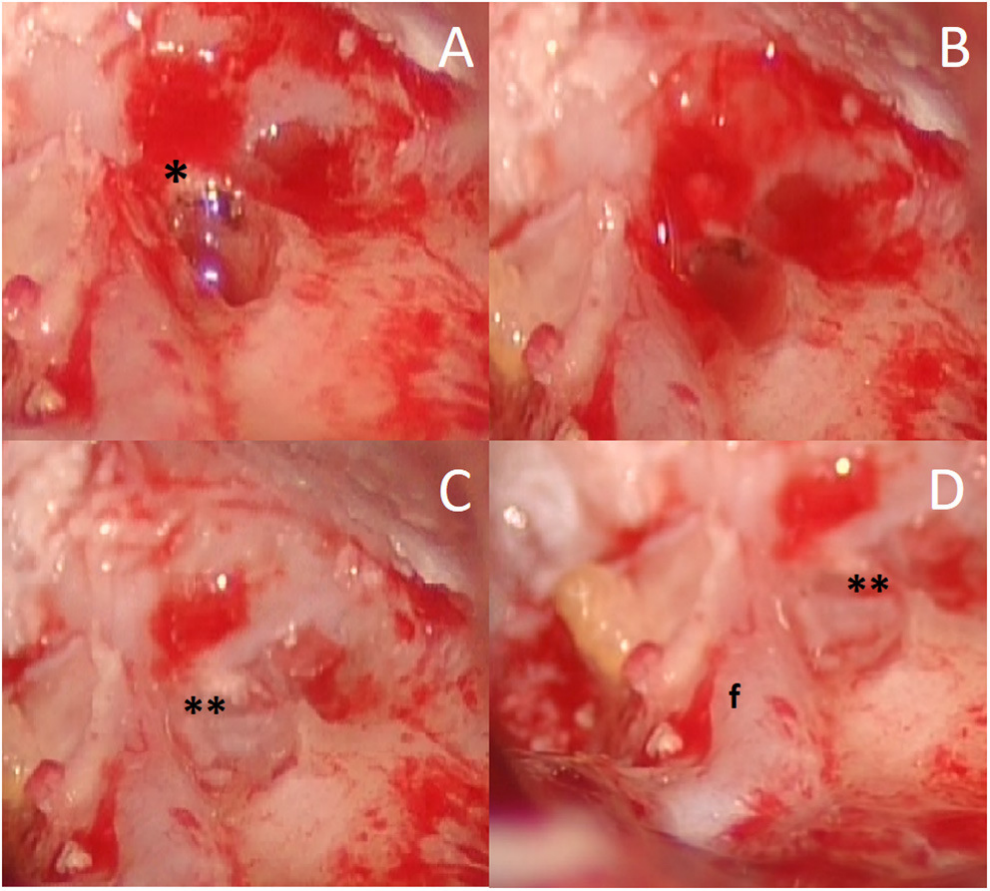
Female, 75 years old, intraoperative view; **(A)** fistula of the lateral and superior semicircular canals (asterisk) after removal of cholesteatoma matrix; **(B)** “underwater,” keeping wet of the membranous labyrinth with Ringer's solution and no suction; **(C)** labyrinth opening is closed using a bone wax (double asterisk); **(D)** relationship between semicircular canals fistula (double asterisk) and facial nerve (f).

Using a cottonoid, the closure of the fistula was then reinforced with bone patè and temporalis fascia. This allowed the preservation of the vestibule, sealing the openings of the semicircular canals (Figure 1).

### Follow-Up

A post-operative follow-up ranged from 1 to 10 years (average of 4 years) and a MRI was performed to assess the signs of recurrence of cholesteatoma and Labyrinthine Fistula (LF).

An evaluation using the Gardner–Robertson classification system was performed at 3, 6, and 12 months follow-up; after the first year of follow-up, they were repeated once a year.

For diagnostic purposes for each patient, we performed the MRI study with Fiesta 3D sequences with the Discovery™ MR750 GE 3.0T system Imaging. This equipment is characterized by excellent gradient homogeneity and exceptional stability. The study was performed with a multichannel head and neck coil (Field of view 20 × 20 cm TR 4 ms; TE 2 ms; and Flip Angle 55°). Images have been processed with MIP (Maximum Intensity Projection) for the visualization of the membranous cochlea – the Axis of the projected unrolled membranous cochlea was seen superimposed on the volume rendered by the MIP image.

## Results

Over the study period, 21 patients (14 males and 7 females, with a median age of 51.5 years and a range of 17–83 years) presented middle ear cholesteatoma with LF in one of their ears (Table 1). The left side was affected in 13 patients, whereas 8 had a right-side lesion. All the evaluated cholesteatomas were acquired; 17 patients underwent primary surgery, while 4 patients had previously undergone an otologic surgery in other structures.

**Table 1:** Patient characteristics.

**Patient**	**Age**	**Sex**	**Previous ear surgery**	**Lf localization**	**D-M classification**	**Pre surgical G-R**	**Post-surgical G-R**	**Recurrence**
1	41	M	None	LSC	IIa	Class II	Class II	None
2	53	F	None	LSC	IIb	Class I	Class I	None
3	25	M	None	LSC-SSC	IIa	Class II	Class II	4 Years
4	67	M	4 Years before	LSC-PSC	IIb	Class I	Class II	None
5	19	F	None	LSC	IIa	Class II	Class II	None
6	64	M	None	LSC	IIa	Class II	Class II	None
7	78	M	None	LSC	IIa	Class II	Class II	None
8	33	F	None	LSC-SSC	IIb	Class I	Class I	3 Years
9	59	F	None	LSC-SSC	IIa	Class I	Class I	None
10	17	M	None	LSC	IIa	Class I	Class I	None
11	27	F	None	LSC	IIb	Class II	Class II	None
12	66	M	None	LSC-PSC	IIa	Class I	Class I	None
13	43	M	2 Years before	LSC	IIa	Class I	Class II	None
14	80	M	6 Years before	LSC	IIa	Class II	Class II	None
15	46	M	None	LSC-SSC	IIa	Class I	Class I	5 Years
16	74	F	None	LSC	IIa	Class II	Class II	None
17	47	M	2 Years before	LSC	IIa	Class I	Class I	None
18	51	M	None	LSC	IIb	Class II	Class II	None
19	83	M	None	LSC	IIa	Class I	Class I	None
20	64	M	None	LSC-PSC	IIa	Class II	Class II	None
21	44	F	None	LSC	IIb	Class I	Class I	None

*M, Male; F, Female; G-R, Gardner–Robertson classification; D-M, Dornhoffer and Milewski; Lf, Labyrinthine fistula; LSC, lateral Semicircular Canal; SSC, Superior Semicircular Canal; PSC, Posterior Semicircular Canal*.

The most common symptom was vertigo in 62% of patients (13/21) and 2 of the latter were positive for Hennerbert's sign.

Hearing impairment was present in 48% of patients (10/21). A pre-operative assessment using the Gardner–Robertson hearing classification showed: 52%, Class I (11/21); 48%, Class II (10/21).

The otomicroscopic exams revealed 7 epitympanic perforations, 5 subtotal or total perforations, 5 normal tympanic membranes associated with swelling of their inferior portion, and 2 cases of canal wall up tympanoplasty: 1 of canal wall down tympanoplasty and 1 radical tympanoplasty.

All patients underwent subtotal petrosectomy, and, in 8 patients, a blind closure of the external auditory canal was performed.

The LF was located in the lateral semicircular canal (LSC) in 100% of the patients. The lesion was of an isolated nature in 67% (14/21), while 19% presented an association with LF of the superior semicircular canal (SSC) (4/21) and 14% (3/21) with LF of the posterior semicircular canal (PSC). According to Dornhoffer and Milewski's classification, 71% presented IIa LF (15/21) and 29% (6/21) had IIb LF.

Post-operatively, the vertigo symptoms reduced, and the vestibular signs (Hennerbert sign) disappeared in 2 months, but the Video Head Impulse Test (vHIT) or caloric tests were not performed.

The post-surgical Gardner–Robertson hearing classification evidenced the following: 43% Class I (9/21); 57% Class II (12/21). The two patients, who passed from class I to class II, had an audiometric difference of <10 dB. One of these two cases was a petrous bone cholesteatoma that involved the internal auditory canal.

The Chi-square with Yate's correction did not show any statistical difference between the pre- and post-surgical hearing outcomes (*p* = 0.7).

The presurgical Pearsons' correlation index between age and hearing class showed an *r* = 0.04. The post-surgical one reported a value of *r* = 0.07.

Sixteen patients underwent ossiculoplasty for functional purposes in a second, non-concomitant surgical step.

**Figure 2:**
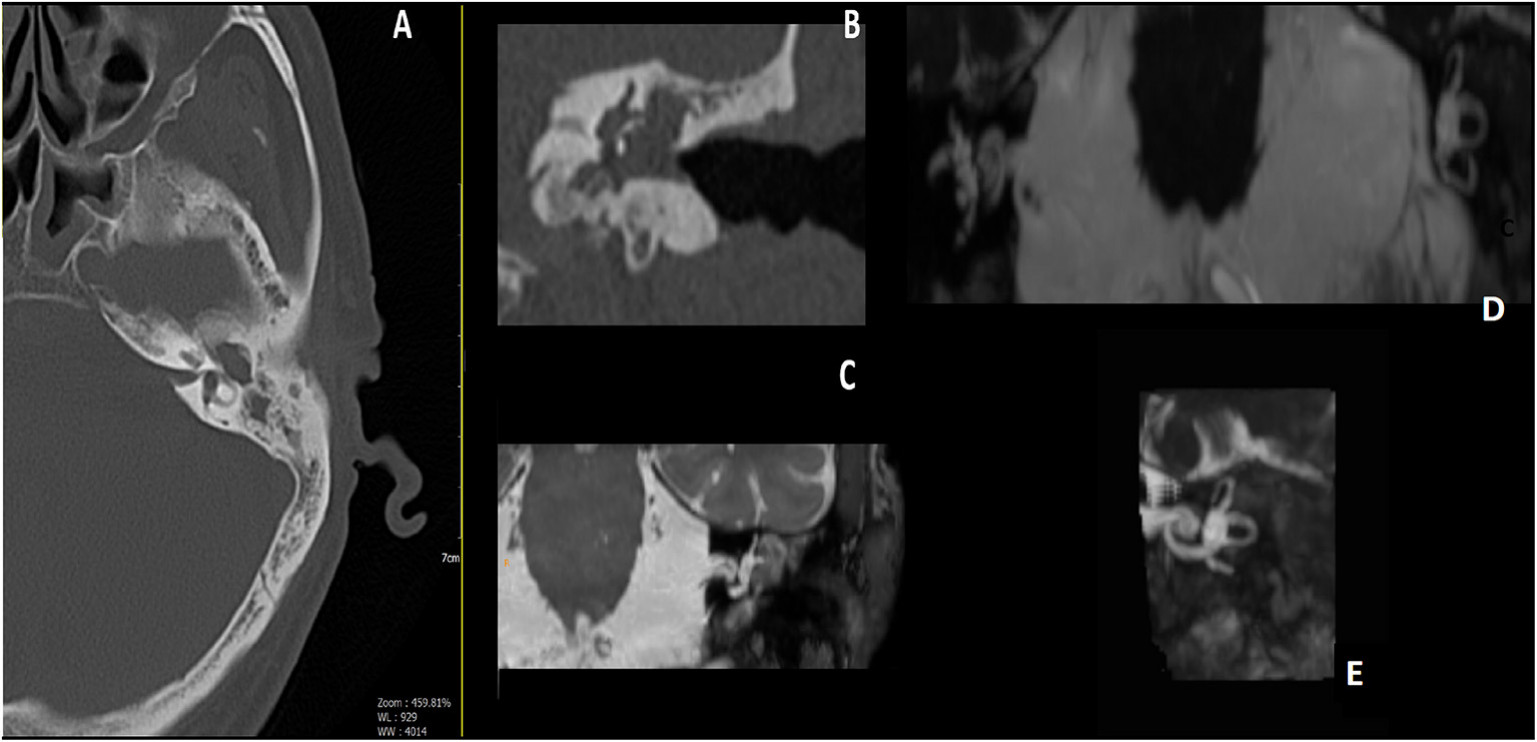
Male, 65 years old; **(A)** pre-operative axial CT scan, erosion of the bony lateral semicircular canal (LSC); **(B)** pre-operative Coronal CT scan, a well-evident erosion of the LSC; **(C)** pre-operative MRI, tissue appears to encase the LSC. **(D)** Post-operative MRI 3D Fiesta 3D sequences (field of view 20 × 20 cm TR 4 ms; TE 2 ms; flip angle 55°); **(E)** post-operative images Maximum Intensity Projection (MIP) for cochlea and semicircular canal visualization, detail of operated side, showing correct visualization of endocanal fluid preservation, and meaning preservation of hearing.

**Figure 3:**
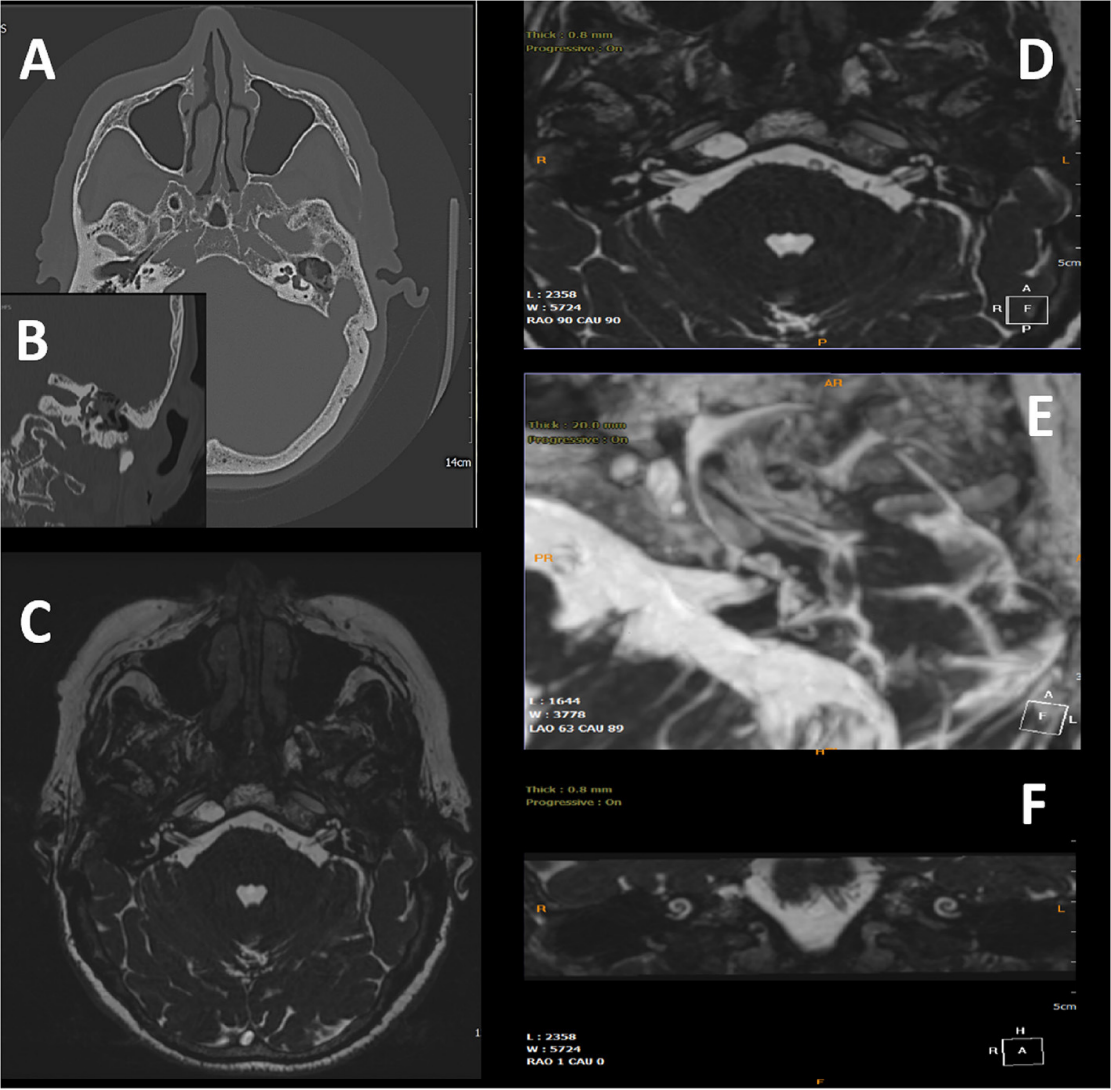
75-year-old woman; **(A)** pre-operative axial CT scan showing the cholesteatomatous tissue and erosion of semicircular canals. **(B)** Particular coronal CT scan showing the erosion of the lateral semicircular canal, and the erosion of the tympanic tegmen; **(C,D)** post-operative images with MRI 3D Fiesta 3D sequences (field of view 20 × 20 cm TR 4 ms; TE 2 ms; flip angle 55°); **(E)** MIP for cochlea and semicircular canal visualization, detail of the operated side shows the correct visualization of the preservation of endocanal fluids. **(F)** MRI Coronal image with preservation of endocanal fluids, meaning preservation of hearing.

**Figure 4:**
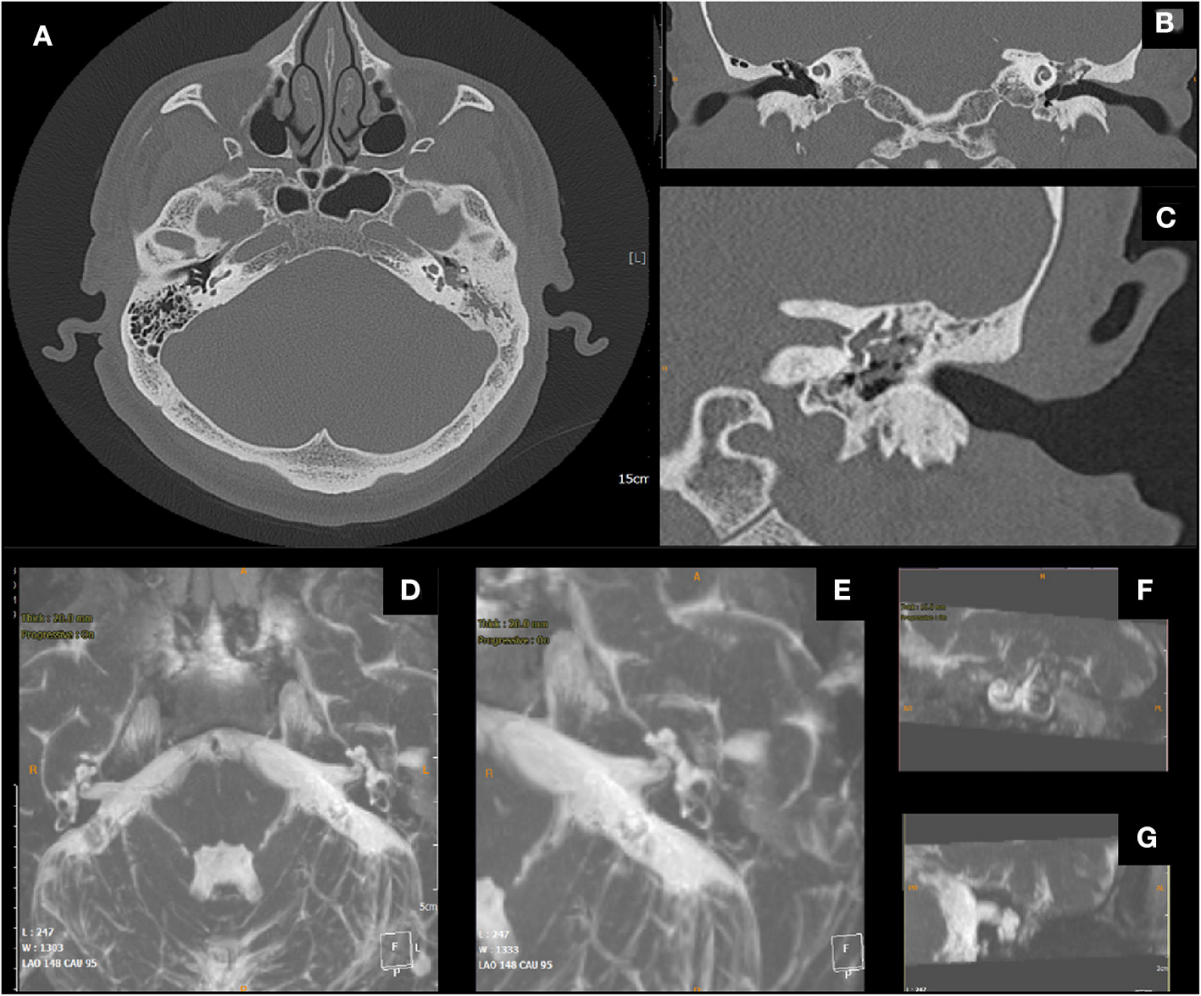
Male, 51 years old; **(A)** pre-operative axial CT scan, cholesteatomatous tissue eroding the wall of the lateral semicircular canal (LSC); **(B)** coronal CT scan, erosion of the LSC; **(C)** detail. **(D)** Post-operative Axial MRI 3D Fiesta 3D sequences (field of view 20 × 20 cm TR 4 ms; TE 2 ms; flip angle 55°); **(E)** post-operative MIP for visualization of the cochlea and the semicircular canals, detail of operated side, proper visualization of endocanal fluid showing its preservation **(F,G)** details.

During the follow-up period, 3 patients (14.2%) showed a limited recurrence at CT imaging. In all the patients, the T2-weighted MR images demonstrated a post-surgical persistence of intralabyrinthine fluids (Figures 2–4).

## Discussion

Labyrinthine fistula is one of the most important complications of cholesteatoma and is defined as an abnormal communication between the inner and the middle ear.

Our data are in line with the current literature that reports an incidence of LF in patients with cholesteatoma ranging from 2.9 to 12.5% with a predominance of LSC, probably owing to its anatomical position (4). Moreover, similar to other studies, the most common symptoms observed were vestibular impairment followed by hearing loss (8).

The presence of LF is usually considered a negative prognostic factor for hearing preservation, and surgical management is still a matter of debate. Moreover, previous papers have made a comparison between different types of fistula, different surgical techniques, and pre-operative levels of hearing status. Therefore, we decided to select a homogenous sample belonging to the Class II Dornhoffer and Milewski LF and the Class I-II Gardner–Robertson, treated exclusively by a partial labyrinthectomy performed by the senior author (GM).

Traditionally, many authors propose leaving the matrix of the cholesteatoma on the LF, thus, reducing the risk of labyrinth damage and hearing loss. On the contrary, others consider that leaving the matrix in the labyrinth leads to a higher risk of infection/inflammation and advocate a radical removal, despite the risk of a dead ear (5).

A recent review reported a hearing preservation rate similar to the staged and un-staged matrix removal (around 90% of hearing preservation), with no statistical difference between the two procedures (9).

The traditional surgical techniques for removal include a canal wall down mastoidectomy (CWDM), a radical cavity surgery (RCS), and a canal wall up mastoidectomy (CWUM). In all cases, after the complete removal of the cholesteatoma, the matrix is peeled from the LF that is covered with fascia and/or fibrin glue, and bone dust. The required major caution is to avoid accidental suction of the perilymph. The risk of recurrence is relatively high.

Thangavelu et al. recently reported their experience with the “underwater technique,” defining it as an evolution of those proposed by Yamauchi and Misale (8, 10, 11). These authors employed a saline solution that is added to corticosteroids and antibiotics to irrigate the surgical area during the matrix removal. However, they peeled the matrix and did not drill the surrounding area, leading to a possible risk of recurrence.

In 2008, Magliulo et al. reported their experience with partial labyrinthectomy in the treatment of LF (5). The technique may be performed in cases of single or multiple fistulae (one or more semicircular canals) when it should have been avoided as the cochlea or vestibule were involved. The key point of partial labyrinthectomy surgery is the preservation of structures by keeping them wet with Ringer's solution throughout the procedures and by not performing suction close to the opened LF. The bony labyrinth is drilled underwater without suction, removing the entire cholesteatoma matrix and quickly plugging the site before and after the LF. This faster plugging of the labyrinth makes it possible to preserve the perilymph and the endolymph fluid and the hearing function (6).

The hearing outcomes obtained, from 2008 to 2020, are in line with all the techniques described with the preservation of pre-operative hearing levels in 90% of the cases. On the other hand, in our cases, there were no cases of the dead ear, and a serviceable hearing was kept in 100% of cases. Moreover, our data also included a Petrous Bone Cholesteatoma (PBC) fistula that reached the internal auditory canal without any hearing post-surgical deficit. This surgical method, in fact, was previously adopted for Schwannoma surgery by Magliulo et al. who modified the technique proposed by McElveen et al. (12).

Also, the partial labyrinthectomy differs from the other techniques for its reduced risk of recurrence that appeared lower than that described by Thangavelu (9 vs. 20%) and is not comparable with Yamauchi and Misale's papers that did not report any pertinent data.

In conclusion, hearing preservation may be possible by employing underwater techniques and by partial labyrinthectomy owing to the ion balance produced by irrigation that allows the preservation of the anatomical structure and the functional activity of the labyrinth. However, to the best of our knowledge, none has radiologically evaluated it. Consequently, we performed a follow-up of the T2 imaging MR to study the labyrinth fluid. The obtained images confirmed the normal anatomical structure and the fluid of the inner ear in all the treated patients.

The limitations of this paper are related to the small sample size of patients, resulting from the choice to select only certain patients with LF. Moreover, tests for vestibular function were not performed. Further multicenter studies with larger samples are underway to confirm the described results.

## Data Availability Statement

The original contributions presented in the study are included in the article/supplementary material, further inquiries can be directed to the corresponding author/s.

## Ethics Statement

The studies involving human participants were reviewed and approved by Sapienza University of Rome. Written informed consent to participate in this study was provided by the participants' legal guardian/next of kin.

## Author Contributions

GM and AP: conceptualization. AMi: methodology. DM: software. GI, SC, and AMa: validation. AP: formal analysis and data curation. VR and CV: investigation. GM: writing—original draft preparation and supervision. CV: writing—review and editing. SC: visualization. All authors have read and agreed to the published version of the manuscript.

## Conflict of Interest

The authors declare that the research was conducted in the absence of any commercial or financial relationships that could be construed as a potential conflict of interest.

## Publisher's Note

All claims expressed in this article are solely those of the authors and do not necessarily represent those of their affiliated organizations, or those of the publisher, the editors and the reviewers. Any product that may be evaluated in this article, or claim that may be made by its manufacturer, is not guaranteed or endorsed by the publisher.
